# Disinfection by the Vapour of Formaldehyde

**Published:** 1896-07-25

**Authors:** H. S. Wansbrough Jones


### MEDICAL PROGRESS AND HOSPITAL CLINICS.

[*The Editor will be glad to receive offers of co-operation and contributions from members of the profession. All letters should be addressed to* THE EDITOR, AT THE OFFICE, 428, STRAND, LONDON, W.C.]

### DISINFECTION BY THE VAPOUR OF FORMALDEHYDE.

By H. S. WANSBROUGH JONES, M.B., C.M.Edin., B.Sc.Lond.

It is a remarkable fact that the English medical papers have almost unanimously neglected to notice the reports of experiments made in France and Germany on the disinfection of rooms by the vapour of formaldehyde. And yet it is certain that this is the only practical method yet discovered of disinfecting rooms without injuring fabrics, destroying colours, or tarnishing metals, and at the same time affording such perfect efficiency in the way of penetration that organisms concealed in mattresses, or beneath considerable thicknesses of woollen materials, are invariably destroyed.

In 1893 Professor Lehrmann published reports of a series of experiments on disinfection by a 40 per cent. solution of formaldehyde, and Dr. Atkinson published in the *Lancet* of December 16th, in the same year, an account of the results he had obtained in the disinfection of houses by the same method. The results were good, but at least 24 hours was needed to complete the process, and it was impossible to ensure a uniform distribution of the vapour. By the methods now in use it is possible to obtain perfect results in a few hours.

Dr. Bardet's method consists in the oxidation of methylic alcohol, Dr. Trillat's in the liberation of the formaldehyde vapour by heating the formalin of commerce—which is, of course, merely a solution of formic aldehyde—in an autoclave with the addition of a neutral salt.

The autoclave may be used either inside or outside the room to be disinfected; but as the vapour is very irritating, and the apparatus needs attending to, it is obviously more convenient to have it outside.

When the pressure in the autoclave has reached from 3 to 4½ atmospheres the vapour is allowed to escape into the room, and it was found by M.M. Trillat and Roux that an autoclave with four jets would in an hour supply enough vapour to saturate a room of more than 500 c. metres volume.

Experiments have been made on the bacillus pyocyaneus, the bacillus coli, the b. prodigiosus, anthrax bacilli, or their spores, tubercle bacilli in sputum or otherwise, and various other organisms. Cultures of the organisms were spread on articles in the rooms or on pieces of cloth, which were placed in all parts of the chamber, under mattresses in pockets, out-of-the-way places being especially chosen in order to test the penetrating power. After thoroughly saturating the room with the vapour of formaldehyde for several hours attempts were made to grow the organisms again after first washing the articles in a solution of ammonia in order to get rid of the formaldehyde.

The results have in all cases been satisfactory. MM. Roux and Trillat, for example, report an experiment in which a room of 1,400 c. metres capacity was tested in this way in which the sterilisation was perfect. Dr. Bosc, of Montpelier, has had equally good results in experiments made in a hospital ward. The same observers have further tested the effects of the vapour on the air and dust in the rooms.

In MM. Roux and Trillat's experiments on the sterilisation of air, 50 litres of air were drawn by an air-pump through bouillon contained in a flat receptacle, so that the bubbles of air would in passing remain for some time in contact with the bouillon, care being taken to neutralise by ammonia any formaldehyde that might pass through, and so exercise a retarding influence on the growth of any organisms that remained. It was found, for example, that where before disinfection there were 120 bacteria, there were none after. The experiments proved also that the dust on the walls could be completely sterilised, though in some cases the sterilisation was not so complete in the dust on the ground. In one case specimens of dust from the sweepings of a house were placed in twelve receptacles in different parts of an inhabited house of three storeys. Ten litres of formaldehyde were used, and the apparatus allowed to work for four hours. Half the specimens used for sowing on bouillon were washed in ammonia. The result was as follows: Ground floor, four specimens; bouillon cloudy after forty-eight hours, in two cases. Room (furthest away), three specimens, bouillon clear in all cases. Room on first floor, one specimen, all the bouillons clear. Room on second floor, four specimens, all clear. There was nothing extraordinary in the fact that two of the specimens on the ground floor were not sterilised, as they were very near a badly fitting door.

As regards penetrating power the results are no less conclusive. Thus, Dr. Bosc found that staphylococci concealed in the pocket of a coat, and colon bacilli placed under a mattress folded on itself, were rendered absolutely sterile. MM. Roux and Trillat have discovered an ingenious method of testing this penetrating power. The action of formaldehyde on gelatine is to render it insoluble; to make use of this property as a test little cubes of glass are coated with liquefied gelatine. When the gelatine has set these are placed in various positions in the room which is being sterilised, and after the process is completed examined by immersion in boiling water. It is found that on those cubes which have been exposed to the action of formaldehyde the gelatine coating is insoluble. Another test used by the same observers depends on the power which formaldehyde possesses of converting aniline reds into blues or violets. Bits of cloth dyed with fuchsine can be used in this way as tests, or a combination of this and the gelatine test can be used, the gelatine being dyed with fuchsine before the glass cubes are coated.

MM. Roux and Trillat further demonstrated that animals can live in an atmosphere that has been treated by formaldehyde vapour, if precautions are taken to first get rid of the vapour. This is done by washing first with a solution of ammonia, and then with sulphuric acid. This treatment would, of course, have no chemical action on any oxides of carbon that might have been formed during the disinfecting process, so that it may be taken as proved that the process is unattended by any risk from the evolution of carbonic oxide. There is yet another point which has been brought out by these experiments on formaldehyde. It has been proved by MM. Pottevin, and also by MM. Roux and Trillat, that to obtain the best results a temperature of about 30 deg. Fahr. is necessary. Still, it was found by actual experiments in rooms that it is possible to completely sterilise walls, ceilings, floor, dust, air, and all contents at the ordinary temperatures. There can be little doubt that from the point of view of preventive medicine, this is one of the most important practical discoveries that has been made for many years. It is to be hoped that some of our medical officers of health, or better still, our Local Government Board, will soon insist on the adoption of these measures in all cases of infected residences.

